# A Comparative Study of Postoperative Complications Associated with Distal Gastrectomy and Pylorus-Preserving Gastrectomy among Gastric Cancer Patients Based on Nationwide Survey Data and Propensity Score Weighting

**DOI:** 10.3390/cancers16122203

**Published:** 2024-06-12

**Authors:** Sang-Ho Jeong, Miyeong Park, Kyung Won Seo, Rock Bum Kim, Jae-Seok Min, Information Committee of the Korean Gastric Cancer Association

**Affiliations:** 1 Department of Surgery, Gyeongsang National University College of Medicine, Gyeongsang National University Changwon Hospital, Changwon 51472, Republic of Korea; jshgnu@gmail.com; 2 Department of Anesthesiology, Gyeongsang National University Changwon Hospital, Changwon 51472, Republic of Korea; ele93547@naver.com; 3 Department of Surgery, Kosin University Gospel Hospital, Busan 49267, Republic of Korea; hahachristi@gmail.com; 4 Regional Cardiocerebrovascular Disease Center, Gyeongsang National University Hospital, Jinju 52727, Republic of Korea; krb747@gmail.com; 5 Department of Surgery, Korea University College of Medicine, and Division of Foregut Surgery, Korea University Anam Hospital, Seoul 02841, Republic of Korea

**Keywords:** gastric neoplasms, postoperative complications, distal gastrectomy, pylorus-preserving gastrectomy, nationwide survey

## Abstract

**Simple Summary:**

This study compared postoperative complication rates between pylorus-preserving gastrectomy (PPG) and distal gastrectomy (DG) using Korean nationwide survey data with propensity score weighting (PSW). PPG preserves gastric function but may lead to more complications than DG. Before PSW, 87.8% of DG and 87.1% of PPG patients had no complications (*p* = 0.053). Severe complications were more frequent in PPG (6.6%) than in DG (3.8%) (*p* = 0.039). After PSW, overall and severe complication rates were similar (*p* = 0.960 and *p* = 0.574). Anastomotic stricture and leakage rates were higher in PPG (2.9% and 1.7%) than in DG (0.6% and 0.5%) before PSW, but not after (*p* = 0.999 and 0.123). The PSW-adjusted analysis indicates no significant difference in complication rates between PPG and DG.

**Abstract:**

Background/Objective. This study aimed to compare complication rates between pylorus-preserving gastrectomy (PPG) and distal gastrectomy (DG) using Korean nationwide survey data and propensity score weighting (PSW). PPG preserves gastric function but may lead to more postoperative complications than DG. Methods and Results. We analyzed 9424 gastric cancer patients who underwent either DG (*n* = 9183) or PPG (*n* = 241). PSW balanced variables such as age, sex, TNM stage, comorbidities, ASA score, and surgical approach. Before PSW, 87.8% of DG patients and 87.1% of PPG patients had no complications (*p* = 0.053). Severe complications (Clavien–Dindo IIIa or higher) were more frequent in PPG (6.6%) than in DG (3.8%) (*p* = 0.039). After PSW, overall complication rates (*p* = 0.960) and severe complication rates (*p* = 0.574) were similar between groups. Incidence rates of anastomotic stricture and leakage were higher in PPG (2.9% and 1.7%) compared to DG (0.6% and 0.5%) (*p* = 0.001 and 0.036) before PSW, but these differences were not significant after PSW (*p* = 0.999 and 0.123). Conclusion. The PSW-adjusted analysis indicates no significant difference in overall and severe complication rates between PPG and DG in gastric cancer patients.

## 1. Introduction

Function-preserving gastrectomy has emerged due to its beneficial postoperative outcomes. Various approaches have been used to perform function-preserving gastrectomy up to the location of the gastric tumor in cases of early gastric cancer (EGC). These techniques can be employed to minimize the extent of gastric resection and improve postoperative functionality without compromising the recurrence rate after surgery [1,2,3,4,5,6,7]. These function-preserving gastrectomy methods include pylorus-preserving gastrectomy (PPG), proximal gastrectomy (PG), vagus nerve-preserving gastrectomy, and sentinel node navigation with stomach-preserving surgery. Among these methods, PPG can be used to resect EGC tumors commonly located in the middle third of the stomach.

PPG was first used to treat peptic ulcers and was later used to treat gastric cancer in Eastern Asia countries Japan and Korea [5,8,9,10]. While some retrospective studies or meta-analyses have highlighted the functional advantages of PPG compared with conventional distal gastrectomy (DG) for EGC, high-quality multicenter randomized controlled trials have yet to be performed, and, therefore, there is no solid evidence regarding the superiority of PPG. Nevertheless, PPG offers several benefits compared to conventional DG, including lower incidence rates of bile reflux, dumping syndrome, and gallstones. Furthermore, PPG offers superior nutritional advantages resulting in minor changes in body weight [8,11,12,13,14,15,16,17].

However, PPG has several disadvantages; therefore, it is not widely used to treat EGC. After PPG, delayed gastric emptying can occur because the remaining pylorus does not function initially, thus leading to complications such as gastric acid reflux [8,18,19,20]. Compared to conventional DG, PPG requires sophisticated surgical techniques to save the pylorus [8,21,22,23,24]. During surgery, accurate localization of the gastric tumor is sometimes difficult [8,25]. When performing lymph node dissection during PPG, additional care is needed, such as saving the right gastric artery and first branch of the gastroepiploic artery [8,21]. However, despite such efforts, the incidence of postoperative complications in the PPG group was not significantly lower than that in the DG group, which is an obstacle to the widespread application of PPG. Selection bias may have affected the results of previous retrospective studies comparing DG and PPG because PPG was mostly performed at the same institution or at a single institution [15,26,27]. In contrast, the present study compared the occurrence of complications between DG and PPG based on data from the 2019 Korean Gastric Cancer Association (KGCA)-led nationwide survey targeting a large number of institutions [28]. Additionally, in this nationwide survey study, propensity score weighting (PSW) was implemented to reduce bias and minimize the difference between the DG and PPG groups.

## 2. Materials and Methods

### 2.1. Collection of Survey Data

The 2019 nationwide survey conducted by the Information Committee of the KGCA utilized a case report form (CRF) [28,29]. The CRF comprised 54 questions and was sent to each institution participating in this nationwide survey. The role of the Information Committee of the KGCA was to review and filter any incorrect or missing data, with representatives from each institution being consulted for clarification. The data collection period spanned from March to December 2020, during which patient demographic data, medical history, pathological findings, operative methods, and surgical outcomes were collected via email [28]. The histological data were categorized according to the 2010 World Health Organization classification, while pathological staging followed the eighth edition of the American Joint Committee on Cancer tumor–node–metastasis (TNM) classification [30,31]. This multicenter, retrospective, observational study included 14,076 patients from 68 institutions. Furthermore, the need for informed consent was waived. Approval for the study was obtained from the Institutional Review Board (IRB) at Gyeongsang National University Hospital in Changwon (IRB No. GNUCH-2023-09-024).

**Figure 1:**
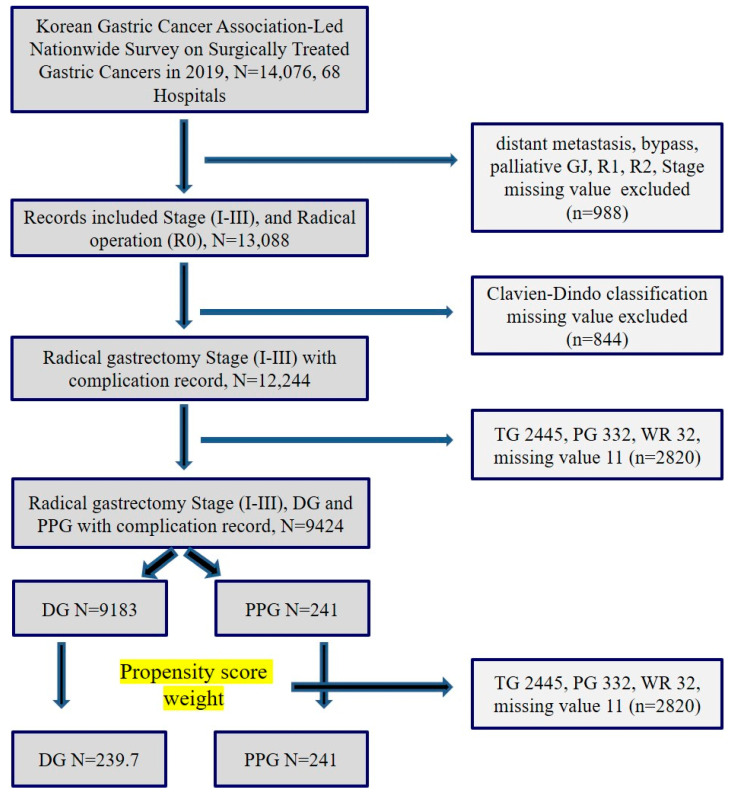
Flow chart of study selection.

We included a total of 13,088 patients who underwent curative radical gastrectomy (R0). To analyze complication rates, we excluded cases with missing complication values, leaving us with 12,244 patients diagnosed with stages 1 to 3 gastric cancer and with Clavien–Dindo (CD) classification complication records [32]. Within this group, we further excluded patients who underwent total gastrectomy (TG) (*n* = 2445), PG (*n* = 332), wedge resection (WR) (*n* = 32), or had missing values (*n* = 11), ultimately resulting in a sample of 9424 patients who underwent either conventional DG (*n* = 9183) or PPG (*n* = 241). PSW was conducted to compare data between the DG (*n* = 239.7) and PPG (*n* = 241) groups (Figure 1). The reasons why some surgeons prefer PPG to conventional DG are several benefits such as lower incidences of dumping syndrome, bile reflux, and gallstones after gastrectomy.

Postoperative complications were events that occurred within 30 days after surgery for gastric cancer, including anastomotic site problems (e.g., stricture or leakage), duodenal stump leakage, intra-abdominal fluid retention or abscess, hemorrhage in the intra-abdominal or intraluminal area, mechanical ileus, wound complications, pancreatic fistula, heart disease, cerebrovascular events, and pneumonia [28]. These complications were categorized using the CD classification. Severe complications were defined as those included in the CD IIIa or higher subgroup. Postoperative death was defined as death within 30 days after gastrectomy or during hospitalization [28].

### 2.2. Statistical Analysis

Categorical variables are presented as frequency (proportion) values and were compared by chi-square tests to test for differences between groups.

Continuous variables are presented as the mean ± standard deviation (SD) or as medians and interquartile ranges (IQRs).

Differences in continuous variables were evaluated using independent sample *t* tests with non-PSW data and weighted independent sample *t* tests with PSW data.

It was necessary to compare the two groups (DG and PPG) with regard to potential confounding variables because there were differences in the confounding variables between the two groups.

This was accomplished using propensity score weighting. The variables used to compute the weight based on the propensity score were age, sex, TNM stage, comorbidities, ASA score, and approach method, all of which differed between the DG and PPG groups.

After conducting PSW, the balance of variables between the two groups was assessed by plotting the love plot, which presents unweighted and weighted standardized mean differences of variables.

Odds ratios (ORs) for severe complications (CD IIIa or higher) were assessed from logistic regression models for non-PSW data and weighted logistic regression models for PSW data.

After multivariate logistic analysis and PSW were performed, the covariates were selected based on clinical considerations and confounders known from risk stratification models.

All *p* values (2 tailed) < 0.05 were considered to indicate statistical significance.

Statistical analysis was performed using the statistical software R 4.3.0 (R Core Team (2021), R Foundation for Statistical Computing, Vienna, Austria. URL https://www.R-project.org/, accessed on 1 February 2024), including the PSW packages “cobalt” and “WeightIt”.

## 3. Results

### 3.1. Comparison of Patient Demographics between the DG and PPG Groups before PSW

The demographic characteristics of the enrolled patients are shown in Table 1. Univariate analysis of the DG and PPG groups revealed that patient age, sex, body mass index, tumor size, TNM stage, presence of comorbidities, ASA score, surgical approach, severe morbidity rate, and severe complication rate were significantly different between the two groups.

The patients in the DG group (average 62.9 years) were significantly older than those in the PPG group (average 60 years) (*p* < 0.001). In terms of sex, the proportion of women was lower in the DG group (35.6%) than in the PPG group (53.5%) (*p* < 0.001). Body mass index (BMI) was greater in the DG group (average 24.1 kg/m^2^) than in the PPG group (average 23.6 kg/m^2^) (*p* = 0.013). The tumor size was greater in the DG group (average 3.3 cm) than in the PPG group (average 2.2 cm) (*p* < 0.001). TNM stage I was less common in the DG group (74%) than in the PPG group (94.2%) (*p* < 0.001). There was a higher rate of comorbidities in the DG group (59.5%) than in the PPG group (37.3%) (*p* < 0.001). The prevalence of an ASA score of 1 was lower in the DG group (23.7%) than in the PPG group (42.7%), while the prevalences of scores of 2 and 3 were higher in the DG group than in the PPG group (*p* < 0.001).

The two operations differed significantly in terms of the approach used (*p* < 0.001). Open surgery was performed in 20.3% of the patients in the DG group but in only 1.2% of the patients in the PPG group. In the case of robotic gastrectomy, DG was performed in 6.6%, and PPG was performed in 25.7%. Similarly, laparoscopic gastrectomy was performed in 73.1% of patients in the DG group (65.2% in the totally laparoscopic group and 7.9% in the laparoscopy-assisted group) and 73.0% of patients in the PPG group (47.3% in the totally laparoscopic group and 25.7% in the laparoscopy-assisted group). There was no significant difference in the number of harvested lymph nodes between the DG group (average 38.8) and the PPG group (average 40.7) (*p* = 0.07).

Before PSW, there was no significant difference in the percentage of patients who experienced postoperative complications (87.8% in the DG group and 87.1% in the PPG group) (*p* = 0.053) (Table 1). On the other hand, when comparing the rates of severe complication (CD IIIa or higher), there was a significant difference between the DG (3.8%) and PPG (6.6%) groups (*p* = 0.039). Even when severe complications were compared among patients who underwent minimally invasive surgery rather than open surgery, there was a significant difference between the DG (3.3%) and PPG groups (6.6%) (*p* = 0.017). However, among patients who underwent minimally invasive surgery, the postoperative mortality rate was not significantly different between the DG (0.2%) and PPG groups (0%) (*p* = 1.0).

### 3.2. Multivariate Analysis of Risk Factors for Severe Complications (CD IIIa or Higher) and PSW

Before conducting PSW, the incidence rates of severe complications (CD IIIa or higher) were compared between the DG and PPG groups. The complications that were significantly different between the two operations were anastomotic leakage and stricture, both of which were more common in the PPG group (*p* = 0.036 and 0.001) (Table 2).

**Figure 2:**
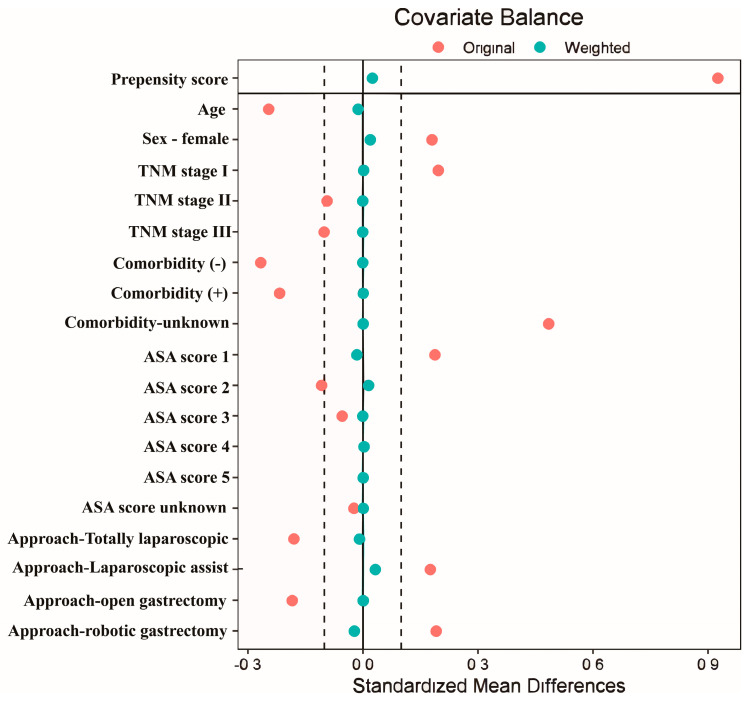
Absolute standardized differences before and after propensity score weighting comparing covariate values between the digital gastrectomy (DG) and pylorus-preserving gastrectomy (PPG) groups. TLG, totally laparoscopic.

After conducting PSW, multivariate logistic regression analysis was performed using severe complications (CD IIIa or higher) as the dependent variable (Table 3). Sex, disease stage, comorbidities, ASA score, and surgical approach were identified as significant risk factors for severe complications. Based on the results of multivariate analysis, the propensity score was calculated, and PSW was performed to adjust weights in the DG group. As a result, compared to the original red spot, the weighted group blue spot after PSW had standardized mean differences gathered around the value of 0, showing that there was no significant difference between the DG and PPG groups (Figure 2).

### 3.3. Comparison of Patients’ Demographic Data between the DG and PPG Groups after PSW

After conducting PSW, there were no significant differences in patients’ age, sex, TNM stage, presence of comorbidities, ASA score, or length of hospital stay (Table 1). Among the patients’ demographic data, BMI and tumor size were significantly different between the DG and PPG groups. The patients who underwent PPG had significantly lower BMIs and smaller tumor sizes than those who underwent DG (*p* = 0.006 and <0.001). The number of harvested lymph nodes was greater in the PPG group (average 40.7) than in the DG group (average 38.5) (*p* = 0.039).

### 3.4. Comparison of Postoperative Complications between the DG and PPG Groups after PSW

After conducting PSW, the rate of complications did not differ between the DG and PPG groups (*p* = 0.960). Additionally, the rates of severe complications (CD IIIa or higher) did not differ between the two groups (*p* = 0.574) (Table 1).

Even for anastomotic leakage—which showed significantly different rates before PSW—there was no significant difference between the DG (*n* = 0.4) and PPG groups (*n* = 4.0) (*p* = 0.123). Additionally, there was no significant difference in the incidence of anastomosis strictures between DG (*n* = 6.2) and PPG groups (*n* = 7.0) (*p* = 0.999) (Table 2).

## 4. Discussion

This study compared rates of complications between patients who underwent conventional DG and those who underwent PPG to treat gastric cancer. PSW was conducted based on nationwide survey data from the 2019 KGCA. There was a significant difference in the incidence of severe complications between the DG (3.8%) and the PPG groups (6.6%) among patients with CD IIIa or higher before conducting PSW (*p* = 0.039). Among the types of complications, anastomotic leakage and stricture were found to be significantly more common after PPG (1.7% and 2.9%, respectively) than after DG (0.5% and 0.6%, respectively) (*p* = 0.036 and *p* = 0.001) before the conducting of PSW. To minimize bias in the comparative analysis, factors showing differences in complications were identified and compensated for using PSW. However, the occurrence of severe CD IIIa or higher complications did not differ between the DG (12.5%) and PPG (16.0%) (*p* = 0.574) groups.

The implementation of health screening initiatives has led to an increase in the incidence of cases of EGC, especially in East Asian countries such as Korea and Japan [28,33,34,35,36,37]. This is a positive development, as early treatment has shown promising oncologic outcomes. As a result, surgeons are now placing equal importance on the postoperative outcomes, including quality of life (QOL), of these patients with early detected EGC in addition to their survival.

PPG is known to cause relatively more gastric dysmotility complications, such as delayed gastric emptying, than other gastrectomies [8,15,18,19]. Delayed gastric emptying, also known as gastroparesis, is a detrimental condition of gastric motility that is defined by a slow emptying of solids without a mechanical intestinal obstruction. Although there are numerous clinical contexts in which delayed gastric emptying can occur, the majority of cases are associated with idiopathic, diabetic, and postsurgical etiologies due to vagus nerve injury [38]. Because vagal nerve injury occurs following abdominal surgery, such as PPG, certain gastroparesis patients experience delayed stomach emptying. Recently, function-preserving gastrectomies that improve quality of life have become relatively popular in an effort to avoid postoperative dumping syndrome and duodenal juice reflux. One such procedure is PPG, which has been used to treat early gastric cancer in the middle third of the stomach [39]. In the early postoperative phase, however, gastric stasis was noted in patients who undergo PPG. It has been accepted that appropriate pyloric function requires both preservation of the right gastric artery and the pyloric branch of the vagus nerve. The vagal supply and the right gastric artery should be preserved during the delicate dissection of the perigastric and suprapyloric lymph nodes [39]. Therefore, a relatively small number of surgeons have performed PPGs. The reasons why surgeons hesitate to choose PPG instead of DG include the limitations in accurate staging before surgery, the technical difficulties encountered in sufficient removal of lymph nodes while preserving the nerves and blood vessels around the pylorus, and the concerns that there may be relatively inferior long-term oncological outcomes after PPG compared to DG, even though the postoperative survival rates have been reported to be similar between PPG and DG [9]. Improving gastric motility after PPG could enhance the efficacy of PPG and promote its use. Consequently, it is difficult to accurately compare surgical data between patients who underwent DG and the small number of patients who underwent PPG. Among the function-preserving gastrectomies, PPG in particular should be performed only if cT1N0M0 is diagnosed before surgery [40,41]. In addition, surgery can be performed when the tumor size is small and the tumor is appropriately located in the middle third of the stomach [8]. This can also be observed in this study, which showed that the tumor size was significantly smaller in the PPG group than in the DG group.

The advantages of a minimally invasive approach also apply to PPG. Compared to the open approach, laparoscopic PPG provides some benefits, such as reduced operative wound pain, less blood loss during surgery, and early recovery [10,23,42,43,44]. Therefore, in most cases, the laparoscopic approach for PPG is commonly performed instead of open surgery. In the present study, minimally invasive gastrectomy was performed more often than open gastrectomy (*p* < 0.001) (Table 1). More specifically, totally laparoscopic (intra-corporeal anastomosis) PPG (47.3%) was performed the most often, laparoscopy-assisted (extra-corporeal anastomosis) PPG and robotic PPG were performed at similar frequencies (25.7%), and open PPG (1.2%) was performed very rarely. During totally laparoscopic PPG, intracorporeal gastro-gastrostomy can be performed in many ways. Compared to extracorporeal reconstruction, intracorporeal anastomosis is reported to be safer and more feasible for minimally invasive PPG [45,46].

In addition, we compared severe complications (CD IIIa or higher) only in the minimally invasive surgery group. Severe complications were found to have occurred in more patients after PPG (6.3%) than after DG (3.3%) before PSW (*p* = 0.017). However, there was no difference found between the PPG (5.2%) and DG group (6.2%) after PSW (*p* = 0.709). Some of the common and serious surgical complications after gastrectomy are anastomosis leakage and stricture. In the present study, before conducting PSW, the incidence rates of severe complications (CD IIIa or higher) were significantly different between the DG and PPG groups. Both anastomosis leakage and stricture were more common after PPG than after DG (*p* = 0.036 and 0.001) (Table 2). The incidence of anastomosis leakage after PPG (1.7%) might be attributed to the suturing of a wider area in this procedure than in B-I anastomosis. On the other hand, the advantage of PPG is that duodenal stump leakage, another serious complication, does not occur. Even before and after conducting PSW, except for anastomosis leakage and stricture, the occurrence of postoperative bleeding, intra-abdominal injury to another organ, wound problems, and other organ diseases were similar between the DG and PPG groups.

There are several limitations in this study. First, because this study was based on survey data, there were some missing values. Second, PPG is not a popular surgical method for gastric cancer; therefore, the number of patients who underwent PPG (*n* = 241) was notably smaller than the number of patients who underwent DG (*n* = 9183). In addition, 91% of PPG operations were performed in three hospitals. The surgeons with a large amount of experience, like those in South Korea, preferred DG to PPG. This difference might be self-explicative when it comes to the choice of the best method for the operative treatment of gastric cancers in a clinical setting. Third, although delayed gastric emptying is known to occur more frequently after PPG, as many cohort studies have already reported, there were no data on delayed gastric emptying in the present study [9,12,13,16,17,18,47,48]. Nevertheless, to the best of our knowledge, this is the first study to compare postoperative complications between conventional DG and PPG using large-scale nationwide survey data with PSW.

## 5. Conclusions

In conclusion, based on the results of a nationwide survey data analysis in which PSW was applied to minimize bias, the occurrence of all grades or severe complications after PPG was similar to that after DG among gastric cancer patients. Additionally, the incidence rates of postoperative anastomotic leakage and stricture, which are thought to be serious complications, were similar after the two operations.

## Figures and Tables

**Table 1:** Comparison of patients’ features before and after propensity score weighting.

Factors		Before PSM DG (*n* = 9183)	Before PSM PPG (*n* = 241)	*p* Value (95% CI)	Factors		After PSM DG (*n* = 239.7)	After PSM PPG (*n* = 241)	*p* Value
Hospital number		64	14						
Age (years)		62.9 ± 11.8	60.0 ± 11.6	<0.001 (1.34~4.37)	Age (years)		60.2 ± 11.6	60.1 ±11.6	0.842
Sex	M	5918 (64.4%)	112 (46.5%)	<0.001	Sex	M	115.9	112.0	0.751
	F	3265 (35.6%)	129 (53.5%)			F	123.8	129.0	
BMI (kg/m^2^)		24.1 ± 3.3	23.6 ± 2.9	0.013 (0.10~0.859)	BMI (kg/m^2^)		24.2 ± 3.3	23.7 ± 2.9	0.006
Tumor size (cm)		3.3 ± 2.2	2.2 ± 1.3	<0.001 (0.83~1.19)	Tumor size (cm)		2.9 ± 1.7	2.4 ±1.4	<0.001
TNM stage	I	6826 (74.3%)	227 (94.2%)	<0.001	TNM stage	I	225.4	227.0	0.999
	II	1265 (13.8%)	10 (4.1%)			II	10.2	10.0	
	III	1092 (11.9%)	4 (1.7%)			III	4.1	4.0	
Existence of comorbidity		5469 (59.5%)	90 (37.3%)	<0.001	Existence of Comorbidity		18.1	18.0	0.999
ASA score	1	2126 (23.7%)	103 (42.7%)	<0.001	ASA score	1	89.4	90.0	
	2	5424 (60.5%)	113 (46.9%)			2	132.2	133.0	
	3	1378 (15.4%)	24 (10.0%)			3	106.2	103.0	0.955
	4	32 (0.4%)	1 (0.4%)			4	108.9	113.0	
	5	7 (0.1%)	0			5	24.1	24.0	
Hospital stay (days)		8.6 ± 6.7	8.3 ±5.5	0.61	Hospital stay (days)		8.6 ± 7.1	8.4 ± 5.6	0.560
Approach methods	Totally laparoscopic gastrectomy	5990 (65.2%)	114 (47.3%)	<0.001	Approach methods	Totally laparoscopic gastrectomy	115.8	114.0	0.866
	Laparoscopy-assisted gastrectomy	726 (7.9%)	62 (25.7%)			Laparoscopy-assisted gastrectomy	53.9	62.0	
	Open gastrectomy	1863 (20.3%)	3 (1.2%)			Open gastrectomy	3.0	3.0	
	Robotic gastrectomy	604 (6.6%)	62 (25.7%)			Robotic gastrectomy	67.1	62.0	
Number of harvested lymph nodes		38.8 ± 16.1	40.7 ± 16.1	0.07	Number of harvested lymph nodes		38.5 ± 15.0	40.7 ± 16.1	0.039
Clavien–Dindo classification	0	8063 (87.8%)	210 (87.1%)	0.053	Clavien–Dindo classification	0	213.1	210.0	0.960
	I	198 (2.2%)	4(1.7%)			I	5.0	4.0	
	II	576 (6.3%)	11 (4.6%)			II	9.1	11.0	
	IIIa	173 (1.9%)	12 (5.0%)			IIIa	9.1	12.0	
	IIIb	106 (1.2%)	2 (0.8%)			IIIb	2.0	2.0	
	IVa	49 (0.5%)	2 (0.8%)			IVa	1.2	2.0	
	IVb	3 (0.0%)	0			IVb	0.0	0.0	
	V	15 (0.2%)	0			V	0.1	0.0	
Severe morbidity rate (Clavien–Dindo ≥ IIIa)		346/9183 (3.8%)	16/241 (6.6%)	**0.039**		Severe morbidity rate (Clavien–Dindo ≥ IIIa)	12.5	16.0	0.574
Severe morbidity rate in minimal invasive surgery group (TLG + LAG + RAG)		239/7320 (3.3%)	15/238 (6.3%)	**0.017**		Severe morbidity rate in minimal invasive surgery group (TLG + LAG + RAG)	12.3	15.0	0.709
Mortality (Clavien–Dindo V)		48/9069 (0.5%)	0/241 (0%)	0.527		Mortality (Clavien–Dindo V)	0.3	1.0	0.999

DG, distal gastrectomy; PPG, pylorus-preserving gastrectomy; PSW, propensity score weight; CI, confidence interval; BMI, body mass index; ASA, American Society of Anesthesiologists; TLG, totally laparoscopic gastrectomy; LAG, laparoscopy-assisted gastrectomy; RAG, robot-assisted gastrectomy. Severe morbidity means Clavien–Dindo classification IIIa or higher. The difference between continuous variables and their means was found using ANOVA testing and post hoc analysis. The difference of categorical variables in their frequency was implemented using chi-square analysis.

**Table 2:** Comparison of severe postoperative complications (CD IIIa or higher) between the DG and PPG groups after propensity score weighting.

Complication (Total *n* = 9424)	Before PSW DG (*n* = 9183)	Before PSW PPG (*n* = 241)	*p* Value	After PSW DG (*n* = 239.7)	After PSW PPG (*n* = 241)	*p* Value
Anastomosis leakage	45 (0.5%)	4 (1.7%)	0.036	0.4	4.0	0.123
Anastomosis stricture	52 (0.6%)	7 (2.9%)	0.001	6.2	7.0	0.999
Duodenal stump leakage	43 (0.5%)	0 (0%)	0.629	1.3	0.0	0.498
Intra-abdominal bleeding	43 (0.5%)	1 (0.4%)	1.000	1.1	1.0	0.999
Intraluminal bleeding	32 (0.3%)	1 (0.4%)	0.575	0.7	1.0	0.999
Pancreatic fistula	17 (0.2%)	1 (0.4%)	0.373	0.3	1.0	0.999
Intra-abdominal abscess	45 (0.5%)	1 (0.4%)	1.000	0.6	1.0	0.999
Fluid collection	127 (1.4%)	1 (0.4%)	0.386	5.7	1.0	0.068
Wound problem	101 (1.1%)	2 (0.8%)	1.000	0.6	2.0	0.999
Mechanical ileus	103 (1.1%)	0 (0%)	0.117	0.9	0.0	0.499
Pneumonia	123 (1.3%)	4 (1.7%)	0.569	1.1	4.0	0.372
CVA	7 (0.1%)	0 (0%)	1.000	0.1	0.0	0.999
Heart problem	23 (0.3%)	0 (0%)	1.000	0.4	0.0	0.999
Others	359 (3.9%)	9 (3.7%)	1.000	7.2	9.0	0.800

DG, distal gastrectomy, PPG, pylorus-preserving gastrectomy, PSW, propensity score weight, CVA, cerebrovascular accident, difference of categorical variables in their frequency was implemented using chi-square analysis.

**Table 3:** Multivariate logistic regression for severe complications (CD IIIa or higher) between the DG and PPG groups after propensity score weighting.

Covariates		OR (95% CI)	*p* Value
Group	DG	–	
	PPG	1.25 (1.05–1.50)	0.012
Age		1.00 (0.99–1.01)	0.431
Sex	Male		
	Female	0.39 (0.32–0.46)	<0.001
Stage	I	–	
	II	0.39 (0.20–0.68)	0.002
	III	0.88 (0.43–1.63)	0.716
Comorbidity	No		
	Yes	0.91 (0.60–1.42)	0.653
	Unknown	1.72 (1.16–2.66)	0.010
ASA score	1	–	
	2	1.49 (1.20–1.85)	<0.001
	3	3.12 (2.30–4.23)	<0.001
	4	0.30 (∞–4.14)	0.589
	5	0.35 (∞–∞)	1.000
	Unknown	0.36 (∞–∞)	0.999
Approach	Totally laparoscopic	–	
	Laparoscopy-assisted	1.54 (1.25–1.89)	<0.001
	Open gastrectomy	3.08 (1.85–4.96)	<0.001
	Robotic gastrectomy	0.64 (0.49–0.82)	0.001

DG, distal gastrectomy, PPG, pylorus-preserving gastrectomy, CI, confidence interval, BMI, body mass index, ASA, American Society of Anesthesiologists.

## Data Availability

Data are contained within the article.
