# Correction: Jeong et al. A Comparative Study of Postoperative Complications Associated with Distal Gastrectomy and Pylorus-Preserving Gastrectomy among Gastric Cancer Patients Based on Nationwide Survey Data and Propensity Score Weighting. *Cancers* 2024, *16*, 2203

**DOI:** 10.3390/cancers16223745

**Published:** 2024-11-06

**Authors:** Sang-Ho Jeong, Miyeong Park, Kyung Won Seo, Rock Bum Kim, Jae-Seok Min, Information Committee of the Korean Gastric Cancer Association

**Affiliations:** 1 Department of Surgery, Gyeongsang National University College of Medicine, Gyeongsang National University Changwon Hospital, Changwon 51472, Republic of Korea; jshgnu@gmail.com; 2 Department of Anesthesiology, Gyeongsang National University Changwon Hospital, Changwon 51472, Republic of Korea; ele93547@naver.com; 3 Department of Surgery, Kosin University Gospel Hospital, Busan 49267, Republic of Korea; hahachristi@gmail.com; 4 Regional Cardiocerebrovascular Disease Center, Gyeongsang National University Hospital, Jinju 52727, Republic of Korea; krb747@gmail.com; 5 Department of Surgery, Korea University College of Medicine, and Division of Foregut Surgery, Korea University Anam Hospital, Seoul 02841, Republic of Korea

**Text Correction**

There was an error in the original publication [1]. “There was no significant difference in the number of harvested lymph nodes between the DG group (average 38.8) and the PPG group (average 40.7) (*p* = 0.007)”.

A correction has been made to 3. Results, 3.1. Comparison of Patient Demographics between the DG and PPG Groups before PSW, 3rd Paragraph, the last sentence should be:

“There was no significant difference in the number of harvested lymph nodes between the DG group (average 38.8) and the PPG group (average 40.7) (*p* = 0.07)”.

The authors state that the scientific conclusions are unaffected. This correction was approved by the Academic Editor. The original publication has also been updated.
